# Granulin A Synergizes with Cisplatin to Inhibit the Growth of Human Hepatocellular Carcinoma

**DOI:** 10.3390/ijms19103060

**Published:** 2018-10-07

**Authors:** Gan Qiao, Huanli Xu, Cong Li, Xiao Li, Ammad Ahmad Farooqi, Yuming Zhao, Xiaohui Liu, Ming Liu, Dimitrios Stagos, Xiukun Lin

**Affiliations:** 1 Department of Pharmacology, School of Basic Medical Sciences, Capital Medical University, Beijing 100069, China; qiagan@mail.ccmu.edu.cn (G.Q.); 13301261635@163.com (H.X.); leeelf@163.com (C.L.); lx18701569602@126.com (X.L.); yumingzhao@ccmu.edu.cn (Y.Z.); liuxiaohui2013@aliyun.com (X.L.); 2 Institute of Biomedical and Genetic Engineering (IBGE), Islamabad 44000, Pakistan; ammadfarooqi@rlmclahore.com; 3 Key Laboratory of Marine Drugs, Ministry of Education, School of Medicine and Pharmacy, Ocean University of China, Qingdao 266003, China; 4 Department of Biochemistry and Biotechnology, School of Health Sciences, University of Thessaly, Volos 38221, Greece; stagkos@bio.uth.gr

**Keywords:** granulin A, cisplatin, anticancer activity, synergistic effect, hepatocellular carcinoma

## Abstract

Cisplatin is one of the most potent chemotherapy drugs widely used for cancer treatment. However, due to resistance and toxicity, the application of cisplatin for the treatment of hepatocellular carcinoma (HCC) is limited. Our previous study has shown that granulin A (GRN A), an anticancer peptide, is able to interact with enolase1 (ENO1) and inhibit the growth of HCC in vitro. In the present study, we studied the synergistic effect of the combination of cisplatin and GRN A for the inhibitory effect on HCC. An 3-(4,5-dimethylthiazol-2-yl)-5-(3-carboxymethoxyphenyl)-2-(4-sulfophenyl)-2H-tetrazolium (MTS) assay and Chou-Talalay approaches revealed that the combination of GRN A and cisplatin displayed potent synergistic effect. The colony formation and cell viability of HCC cells were inhibited significantly in cells treated with the combination of cisplatin and GRN A, compared with cells treated with cisplatin or GRN A alone. Overexpression of ENO1 diminished the synergistic effect of GRN A and cisplatin in HCC cells. The combination of the two drugs exhibited a more obvious inhibitory effect on cancer cell apoptosis, as analyzed by the cytometry flow, mitochondrial membrane potential (MMP) and western blot analysis. An in vivo study confirmed that the combined use of the two drugs displayed more potent antitumor activity compared to mice treated with cisplatin and GRN A alone; the inhibitory rate of tumor growth was 65.46% and 68.94%, respectively, in mice treated with GRN A and cisplatin. However, the inhibitory rate increased to 86.63% in mice treated with the combination of the two drugs. This study provides evidence that the combination of GRN A and cisplatin is able to sensitize the liver cancer to cisplatin, and that targeting ENO1 is a promising approach for enhancing the antitumor activity of cisplatin.

## 1. Introduction

Hepatocellular carcinoma (HCC) is the third most common cause of cancer-associated mortality worldwide [1,2,3]. There are more than 250,000 new HCC cases and an estimated 600,000 HCC mortalities each year worldwide [1,4]. The prognosis of HCC is very poor, and the five-year survival rate worldwide remains low [5]. Although the combination of surgery and chemotherapy has increased the survival time of patients with HCC in recent years, a significant number of patients still relapse, mainly because of a high potential for vascular invasion, metastasis, drug resistance, and recurrence even after surgical resection [6]. Therefore, developing novel strategies for the treatment of HCC is important.

Cisplatin, an anti-cancer chemotherapeutic agent widely used clinically, can inhibit the replication of DNA [7] and then inhibit the RNA and protein synthesis at high concentrations [8]. The main acting sites of cisplatin are the purine and pyrimidine bases of DNA. Thus far, it has been demonstrated that cisplatin has a strong broad-spectrum anti-cancer effect. It is used for the treatment of ovarian cancer, prostate cancer, testicular cancer, lung cancer, nasopharyngeal cancer, esophageal cancer, malignant lymphoma, and thyroid cancer, as well as HCC [6,9,10,11,12,13,14,15,16]. However, the severe emetic side effect and drug resistance limits its efficacy in the treatment of HCC [17,18,19,20]. In recent years, combination therapy of cisplatin with other anticancer agents have been developed as novel therapeutic strategies for many human cancers [16,21,22]. 

Granulin A (GRN A), a peptide with a molecular 6-kDa, is derived from proteolysis of progranulin (PGRN) [23]. Previous study in our laboratory has shown that GRN A is able to inhibit cancer cell growth significantly [24]. Our further study revealed that the interaction between GRN A and enolase 1 (ENO1) is responsible for the growth inhibitory effect of GRN A on human hepatocarcinoma cells HepG2 [25]. A recent study by Qian et al. reported that the increased expression of the glycolytic enzyme ENO1 is responsible for the cisplatin-resistance in gastric cancer cells [26,27]. Depletion of ENO1 by small interfering RNA (siRNA) significantly reduced glycolysis and reversed drug resistance. It is conceivable that GRN A possesses the ability of synergizing with cisplatin to inhibit cancer growth. In the present study, we investigated the synergistic effect between GRN A and cisplatin. Our results confirmed that GRN A enhances the inhibitory effect of cisplatin significantly; treatment of the hepatocellular carcinoma with the combination of GRN A and cisplatin leads to more potent inhibitory effect on cancer cells growth, as analyzed by apoptosis and colony formation assay. An in vivo study also confirmed that GRN A synergizes with cisplatin to inhibit the growth of HCC in xenograft mice. 

## 2. Results

### 2.1. GRN A Enhanced the Cytotoxicity of Cisplatin Significantly

An MTS assay was used to evaluate the cytotoxicity of several chemotherapeutic agents in the presence or absence of GRN A in HepG2 cells. As shown in Table 1, the half inhibitory concentration (IC_50_) values of cisplatin, cyclophosphamide, paclitaxel, 5-fluorouracil, methotrexate, and gemcitabine were 99.54 ± 7.27, 33.50 ± 3.29, 1.54 ± 0.37, 96.51 ± 8.77, 5.17 ± 0.47, and 7.05 ± 0.93 μM, respectively. In the presence of GRN A (10 μM), the IC_50_ values were decreased to 51.32 ± 5.41, 33.44 ± 3.86, 1.49 ± 0.17, 90.18 ± 6.52, 4.64 ± 0.18, and 6.25 ± 0.79 μM, respectively, for cisplatin, cyclophosphamide, paclitaxel, 5-fluorouracil, methotrexate, and gemcitabine. This data indicated that GRN A significantly enhances the cytotoxicity of cisplatin in HepG2 cells, and that there is a potential synergistic effect between GRN A and cisplatin in HepG2 cells. 

### 2.2. Synergistic Effect of Cisplatin and GRN A in HCC Cells

**Figure 1:**
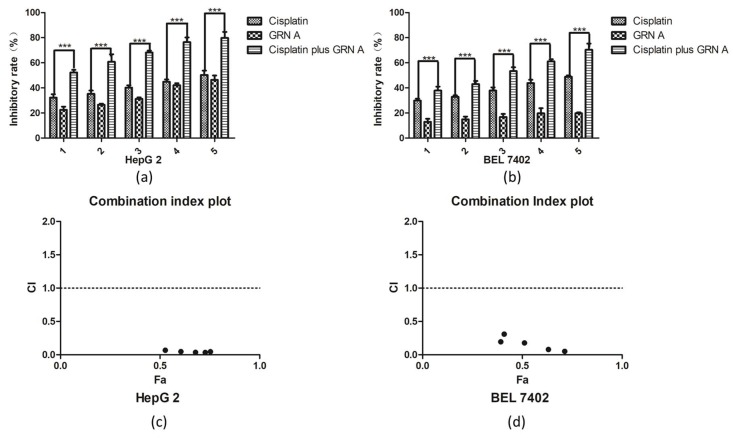
GRN A synergized with cisplatin to inhibit cancer cell proliferation. Cytotoxicity of GRN A, cisplatin, and the combination of the two drugs. HepG2 (**a**) and BEL7402 (**b**) cancer cells were plated on 96 plates. After an incubation for 48 h, cells were treated with certain concentrations of GRN A, cisplatin, and the combination of the two drugs. The inhibitory rate of the cell growth was analyzed by an MTS assay, as described in the materials and methods section. (**c**, **d**) Combination index (CI) Plot. A CI of GRN A and cisplatin was analyzed using the Chou-Talalay approach. CI values were plotted as a function of the fractional affection (Fa) from 0.39 to 0.85 in HepG2 and BEL7402 cells. CI > 1.3, antagonism; CI 1.1–1.3, moderate antagonism; CI 0.9–1.1, additive effect; CI 0.8–0.9, slight synergism; CI 0.6–0.8, moderate synergism; CI 0.4–0.6, synergism; CI < 0.4, strong synergism. (*t*-test, *n* ≥ 3 *** *p* < 0.001)

We performed isobolograms of the drug combination analysis to evaluate the synergistic effect of the two drugs. The results showed that when treating HepG2 cells with 10 μM GRN A, the concentration of cisplatin needed to reach the IC_30_, IC_50_, IC_70_ was 1.55, 19.36, and 28.48 μM, respectively. On the other hand, when treating HepG2 cells with 8 μM GRN A, the concentration of GRN A needed to reach the IC_30_, IC_50_, IC_70_ was 1.95, 28.36, and 33.92 μM, respectively (Figure S1); all of the points fell into the lower part of the solid lines (Figure S1), suggesting that there is a synergistic effect between GRN A and cisplatin. We then used the Chou-Talalay method to further evaluate the synergistic effect of GRN A and cisplatin. The inhibitory rates of cisplatin, GRN A, and the combination of cisplatin with GRN A were analyzed using an MTS assay in HepG2 and human hepatocarcinoma cells BEL7402. As shown in Figure 1a,b, GRN A displayed a synergistic effect with cisplatin at various combinations of cisplatin in both HepG2 and BEL7402 cells. The combination index values were calculated by the Compusyn software (Compusyn Inc, Paramus, NJ, USA). The dose-effect parameter (Dm and r) of the two drugs, either as single agent or in combination, as well as the combination index (CI) values of combinations at IC_50_ were shown in Table 2. The Dm value corresponds to the concentration of drugs needed to induce 50% of cell killing. The r values were higher than 0.95 in the experiments, indicating a good conformity of the dose-effect data. Figure 1c,d show the plots of the combination index for the interaction between the two drugs following the treatment schedule, as described in the materials and methods section. The X axis represents the ratio of the effect on the inhibition of cell growth with combined cisplatin and GRNA. As shown in Figure 1c,d, all the CI values were less than 0.4, suggesting that there is a strong synergistic effect between GRN A and cisplatin in both HepG2 and BEL7402 cell lines. (t-test, * *p* < 0.05; ** *p* < 0.01)

### 2.3. GRN A Synergized with Cisplatin to Inhibit Cancer Cell Viability and Colony Formation

**Figure 2:**
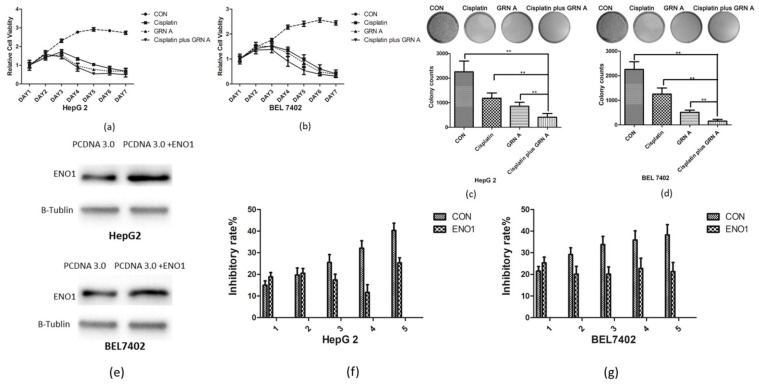
The combination of cisplatin and GRN A inhibited the growth of HCC more obviously than that cisplatin or GRN A alone. (**a**,**b**) Time inhibitory effect of GRN A, cisplatin and the combination of the two drugs on the cancer cells. Cancer cells were treated with cisplatin, GRN A and the combination of the two drugs. The cell viability was analyzed by MTS assay as described in the materials and methods section. (**c**,**d**) The combined application of cisplatin and GRN A inhibited the colony formation significantly in HCC cells. Cells were treated with cisplatin, GRN A, and the combination of the two drugs, and the colony formation was analyzed as described in the materials and methods section. (**e**) Overexpression of ENO1 in HCC cells. (**f**,**g**) HCC cells were transfected with ENO1 for 24 h and treated with combination of two drugs for another48 h. (*n* ≥ 3, ** *p* < 0.01)

To investigate the effect of cisplatin, GRN A, and the combination of cisplatin with GRN A on the growth of HCC cells, cell viability and colony formation assays were performed. As shown in Figure 2a,b, cisplatin or GRN A alone inhibited HCC cell proliferation. Moreover, the combination of cisplatin and GRN A displayed more potent suppressed effect on the proliferation of HCC cells in a time-dependent manner. A colony formation assay revealed that the combination of cisplatin and GRN A exhibited a more obvious inhibitory effect on the colony formation than using cisplatin alone; the colony number was 1185 ± 104 in cells treated with cisplatin alone, while the number reduced to 414 ± 39 in cells treated with the combination of the two drugs in HepG2 cells. Similar results were also found in BEL7402 cells treated with the combination of GRN A and cisplatin (Figure 2c,d). These results suggest that the combined application of GRN A and cisplatin displayed more obvious inhibitory effect of colony formation in HCC cells. In order to evaluate whether synergistic effect is associated with the ENO1 expression, we constructed ENO1 overexpression cells (Figure 2e). Our results showed that the inhibitory effect of cancer cells growth was diminished significantly in cells with an overexpression of ENO1 in both BEL7402 and HepG2 cells, compared with that in the parent cells (Figure 2g,f). These results suggest that the synergistic effect of GRN A and cisplatin can be attributed to the inhibiting of ENO1 by GRN A.

### 2.4. GRN A Potentiated the Effect of Cisplatin Induced Apoptosis

**Figure 3:**
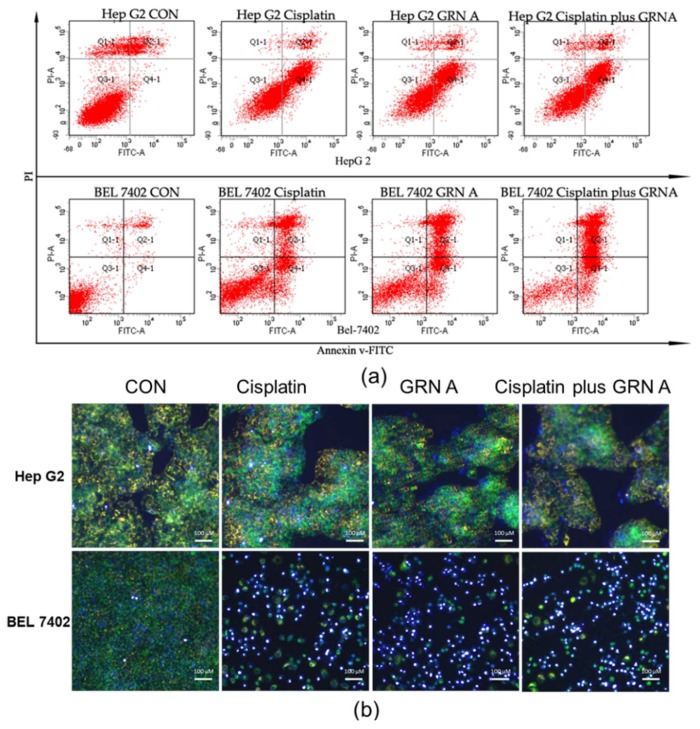
GRN A sensitized HCC cells to cisplatin-induced apoptosis. (**a**) Flow cytometry analysis. Cells were treated with cisplatin (33.33 µM), GRN A (14.57 µM) and the combination of the two drugs for 24 h. Apoptotic intensity was analyzed by flow cytometry as described in the materials and methods section. (**b**) MMP was analyzed by using a MITO-ID probe and 4’,6-diamidino-2-phenylindole (DAPI) double staining approach. The blue color indicated the location of nuclei, while the orange color presented the MMP augmentation. Depolarized effect was indicated by the green fluorescence. Scale bar, 100 µm.

**Figure 4:**
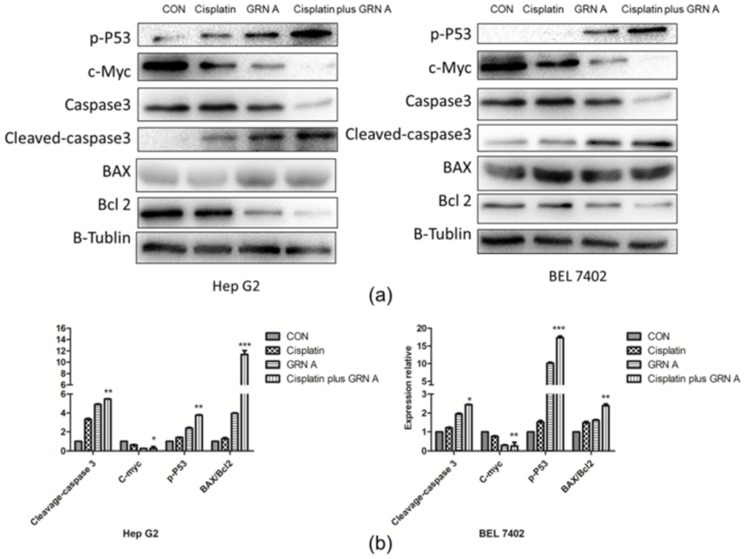
Effects of cisplatin, GRN A, and the combination of the two drugs on the expression of apoptotic related-proteins. (**a**) Western blot analysis. Cells were treated with cisplatin, GRN A, and the combination of the two drugs. The expression of some apoptotic genes, including *BAX*, *Bcl-2*, *c-Myc*, *caspase-3*, and *phosphated-p53*, were determined by western blot, as described in the materials and methods section. (**b**) Quantitative results of gene expression. (*n* ≥ 3, * *p* < 0.05, ** *p* < 0.01, *** *p* < 0.001).

The induced apoptosis of GRN A, cisplatin, and the combination of the two drugs was investigated by flow cytometry, mitochondrial membrane potential, and western blot in HepG2 and BEL7402. Both of the HepG2 and BEL7402 cells were treated with cisplatin (33.33 µM), GRN A (14.57 µM), and their combination for 24 h. After double staining with recombinant annexin V conjugated to green-fluorescent FITC dye (Annexin V FITC) and propidium iodide (PI), a flow cytometry analysis was performed. As shown in Figure 3a, the apoptotic rate is 48.35 ± 2.24%, 49.20 ± 2.74%, and 56.44 ± 5.77%, respectively, in HepG2 cells treated with cisplatin, GRN A, and the combination of the two drugs. Similar results were also found in BEL7402 cells treated with cisplatin, GRN A, and the combination of the two drugs. The change of MMP was also analyzed using a MITO-ID fluorescent probe staining approach. As shown in Figure 3b, the cells treated with the combination of GRN A and cisplatin displayed more obvious change of MMP compared to cells treated with GRN A and cisplatin alone; the orange color nearly disappeared when treated with the combination of the two drugs. Additionally, the western blot analysis revealed that co-treatment with cisplatin and GRN A increased the expression of phosphorylated p53, cleavage caspase 3, as well as the ratio of BAX/Bcl2, and down-regulated the expression of c-Myc in both HepG2 and BEL7402 cells. Moreover, the combined treatment potentiated the effect significantly (Figure 4a,b). These results indicated that the combination of GRN A and cisplatin increased the apoptotic effect in HCC cells.

### 2.5. The Combinationof Cisplatin with GRN A Potentiated the Antitumor Effec in Vivo in HepG2 Xenograft Model

**Figure 5:**
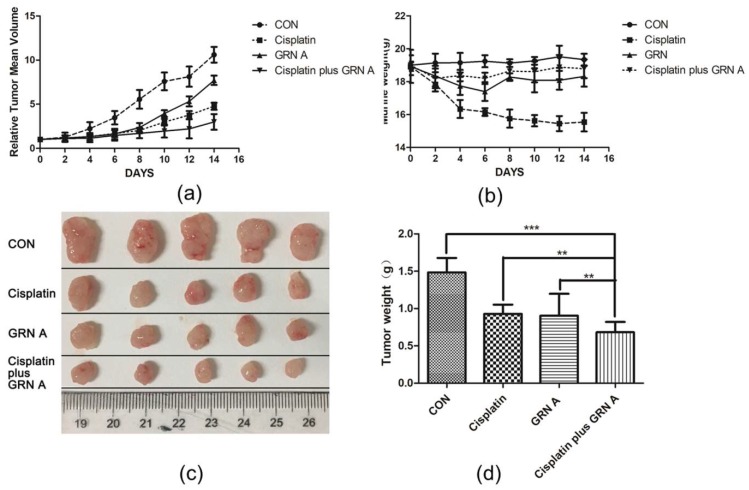
GRN A synergized with cisplatin to inhibit tumor growth in the xenograft model. Mice bearing HepG2 cancer cells were treated with cisplatin, GRNA, and the combination of the two drugs. Tumor volume (**a**) and mouse body weight (**b**) were measured every two days. (**c**) Tumors were excised and photographed after treated with drugs for 14 d. (**d**) Quantitative analysis of tumor weight after treating the mince with drugs for 14 days. ** *p* < 0.01, cisplatin *vs* cisplatin plus GRN A; ** *p* < 0.01, GRN A *vs* cisplatin plus GRN A. (*t*-test, *n* = 5, ** *p* < 0.01, *** *p* < 0.001)

The synergistic effect of GRN A and cisplatin in vivo was evaluated using the xenograft model. Cisplatin, GRN A, and the combination of the two drugs were administrated through an intraperitoneal injection (IP) one time every two days, for 14 days. The tumor volume and mice body weight were measured. As shown in Figure 5, both cisplatin and GRN A were able to inhibit the tumor growth significantly. Furthermore, the combination of cisplatin and GRN A displayed more obvious inhibition on tumor growth as compared to those treated with cisplatin and GRN A alone; the inhibitory rates of tumor growth was 65.46% and 68.94%, respectively, in mice treated with GRN A and cisplatin for 10 days, while the inhibitory rate increased to 86.63% in mice treated with the combination of GRN A and cisplatin. The mean tumor weight was 0.92 ± 0.16 and 0.85 ± 0.12g in mice treated with cisplatin and GRN A, respectively, while the tumor weight reduced to 0.65 g in mice treated with the combination of cisplatin and GRN A. Importantly, the body weight of the mice was less affected in mice treated with the combination of the two drugs, compared with those treated with cisplatin alone. These results indicate that there is a strong synergistic effect between cisplatin and GRN A. Additionally, GRN A synergized with cisplatin increased the antitumor activity in vivo. (t-test, * *p* value < 0.05; ** *p* value < 0.01)

### 2.6. Representative H&E Staining and IHC of the Initial Tumor

**Figure 6:**
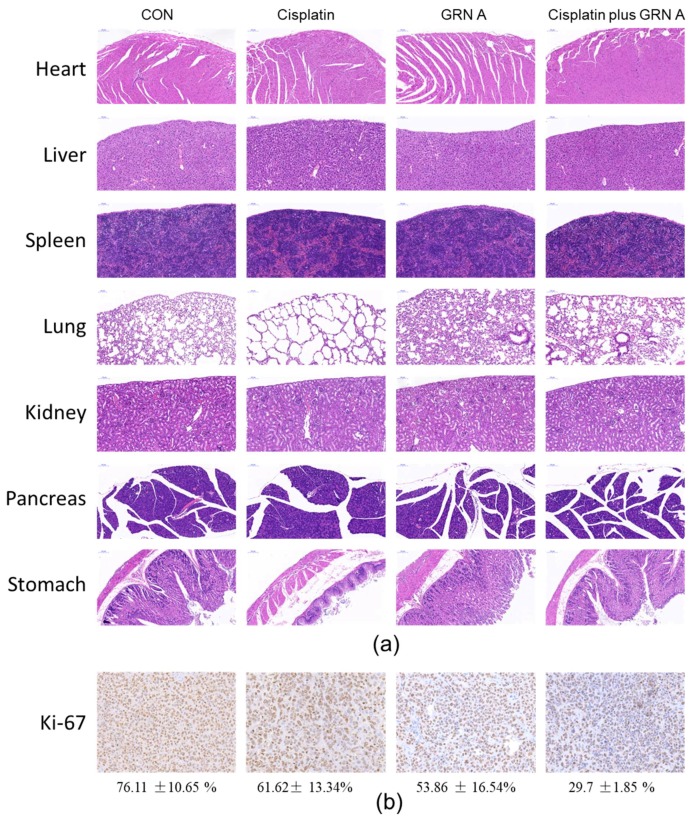
Histological analysis of seven principal organs excised from HepG2 tumor-bearing mice (**a**), and immunohistochemically (IHC) analyses of tumors (**b**) belonging to untreated group, cisplatin-treated (5 mg/kg), GRN A-treated (50 mg/kg) and combination of cisplatin with GRN A. Scale bar, 100 µm.

In order to further evaluate the toxicity of the combination of GRN A and cisplatin on nude mice, hematoxylin and eosin (H&E) staining and immunohistochemistry (IHC) immunologic analysis were performed. As shown in Figure 6a, there is no obvious lesson in the main organs of mice treated with saline or GRN A. However, treated with cisplatin resulted in systematic toxicity in the tissue of lung, stomach, and kidney. Interestingly, no obvious lessons were found in the main organs of mice treated with the combination of cisplatin and GRN A. IHC immunological analysis also revealed that GRN A could sensitize the tumor to cisplatin; the brown particle presented the cellular proliferation marker (Ki-67) expression, as shown in the Figure 6b, and the manual counting data for Ki-67 was performed as described by Yeo, M.K., et al. [28]. IHC staining showed that the positive ratios of the Ki-67 protein in the cisplatin plus the GRN A group were 29.7 ± 1.85%, which is less than the control (76.11 ± 10.65%), cisplatin (61.62 ± 13.34%), and GRN A (53.86 ± 16.54%) groups (*P* = 0.003 and *P* = 0.002, respectively). (t-test, ** *p* < 0.01)

## 3. Discussion

Cisplatin-based therapy is one of the most important chemotherapeutic treatments for cancer. However, its efficacy is greatly limited by drug resistance and undesirable side effects. Therefore, it is of great importance to develop chemosensitizing agents to cisplatin. Over the years, various cisplatin-based combination therapies have been developed. Studies have shown that ginsenoside Rg3, a Glycosides from *Panax ginseng C.A.Mey*, displays potent synergistic antitumor effect with cisplatin in bladder tumor cancer cells [29]. Combination treatment of T24R2 cancer cells results in increased apoptosis via enhancing the expression of cytochrome c, caspase3, and downregulating the expression of Bcl-2 [29]. Zhang Y et al. reported that the antitumor effect of cisplatin was greatly increased by methylseleninic acid [30]. Treatment of cancer cells with the combination of cisplatin and methylseleninic acid induced intracellular oxygen stress, resulting in DNA damage in cancer cells. Additionally, a recent study by Xia, A. et al. [31] confirmed that the co-treatment of non-small lung cancer cells with BEZ235 and cisplatin displayed an obvious synergistic effect, and an increased inhibitory effect on cisplatin-resistant cancer cells A549 was observed. The combination of cisplatin and BEZ235 inhibited the PI3K/AKT/mTOR pathway signaling significantly [31,32]. In the present study, we confirmed that GRN A, a polypeptide, was able to sensitize the antitumor effect of cisplatin both in vitro and in vivo. This study provided more evidence that the combinational therapy of cisplatin is a useful approach for the treatment of cancer.

Granulins (GRNs) are a family of about 6-kDa peptides derived from proteolysis of progranulin (PGRN) [23]. GRNs play an important role in mammalian cell growth, acting both agonistic and antagonistic in cell development [33]. GRN A is initially found and purified from human leukocytes and rat bone marrow [34], and the peptide was confirmed to display proliferation inhibition on human epidermoid carcinoma A431 cells and breast cancer MD-MBA-468 cells [35,36]. Our previous study also showed that GRN A was able to inhibit several cancer cell growth [24,25]. GRN A is also capable of inhibiting migration and invasion of HepG2 cancer cells by interacting with ENO1 [25]. In the present study, we found that GRN A displayed potent anticancer effect in vivo; treatment of the mice bearing hepatocellular carcinoma HegG2 cancer cells with GRN A resulted in a significant inhibitory effect on tumor growth (Figure 5). These results revealed that GRN A has the potential to be developed as a novel anticancer agent.

Studies have revealed that high level of ENO1 was strongly linked to poor prognosis of cancer patients. ENO1 has been proposed to be a potential tumor biomarker of chemo-resistance and overall prognosis [27,37]. Previous studies have shown that down-regulation of ENO1 can increase the sensitivity of cisplatin in gastric cancer cells [38,39]. Our previous study confirmed that the inhibitory effect of GRN A on cancer cells is associated with the interaction between GRN A and ENO1 [25], and GRN A is capable of inhibiting the function of ENO1. In the present study, we found that overexpression of ENO1 attenuated the synergistic effect of GRN A and cisplatin. The results provide further evidence that targeting ENO1 is a promising approach for enhancing the anticancer effect of cisplatin.

## 4. Materials and Methods 

### 4.1. Drugs and Reagents

Cisplatin, cyclophosphamide, paclitaxel, 5-fluorouracil, methotrexate, and gemcitabine were purchased from Sigma-Aldrich (St. Louis, MO, USA). MTS was purchased from Promega Bioscience, LLC. (Madison, WI, USA). The whole cell protein extraction kit and sodium dodecyl sulfate were obtained from Beyotime, lnc. (Shanghai, China). The primary antibodies and secondary antibodies were products of Cell Signaling Technology, Inc. (Boston, MA, USA). The MMP assay kit was purchased from Enzo Life Sciences, Inc. (Farmingdale, NY, USA). All other chemicals used were of the highest purity available from commercial sources.

### 4.2. Cell Culture

BEL7402 cells were purchased from National Infrastructure of Cell Line Resource (Shanghai, China). HepG2 cells were purchased from ATCC (Manassas, VA, USA). BEL7402 cells were cultured in a Dulbecco’s Modified Eagle Medium (DMEM)(Hyclone, lnc. Carlsbad, CA, USA) supplemented with 10% fetal bovine serum and 1% penicillin-streptomycin in a humidified atmosphere with 5% CO_2_ in air at 37 °C. HepG2 cells were cultured in a DMEM medium (Hyclone, lnc. Carlsbad, CA, USA), supplemented with a 10% fetal bovine serum and 1% penicillin-streptomycin in a humidified atmosphere with 5% CO_2_ in air at 37 °C.

### 4.3. Preparation of GRN A

GRN A was prepared in our laboratory as described previously [24]. Briefly, the recombinant GRN A was expressed in bacterial expression systems. Prior to purification, the cells were collected by centrifugation and were sonicated for 10 min. After that, the supernatant containing GRN A was filtered through 0.22 μm filters (Millipore, Bedford, MA, USA). His-tagged GRN A was purified with nickel nitrilotriacetic acid affinity chromatography using self-packed columns in the flexible and intuitive chromatography system (GE Healthcare, IL, USA). The supernatant, containing recombinant GRN A polypeptide, was dialyzed to remove salt in ddH_2_O, and lyophilized.

### 4.4. Cell Viability Assay and Caclulation of CI

Cisplatin, cyclophosphamide, paclitaxel, 5-fluorouracil, methotrexate, and gemcitabine were prepared as previously reported [40]. The GRN A stock solution was prepared by dissolving 20 mg/mL in distilled water. The stock solution for each drug was sterilized with a 0.2 μm syringe filter, aliquoted, and stored at −20 °C until used. The effects of GRN A and cisplatin on cell proliferation were detected by the MTS assay as described previously. IC_50_ was determined based on absorbance readings. The fixed ratios of drug combination were based on the ratios of IC_50_. Briefly, HepG2cells (5 × 10^3^) and BEL7402 cells (3 × 10^3^) were seeded into 96-well plates per well, respectively, and allowed to attach for 24 h before treatment. The cells were treatment to various doses of cisplatin (4.17–66.67 µM), GRNA (1.82–29.07 µM) and the combination of cisplatin with GRN A for 48 h. After that, MTS was added to each well and incubated for another 2 h. Then, the optical density (OD) value was analyzed with an ELISA reader at a wave length of 490 nm.

The synergistic effect of the two drugs was analyzed using isobolograms of the drug combination, as previously reported [41], and the multiple drug-effect equation was used to calculate the inhibitory rate. The interaction between two drugs was also analyzed by the median-effect principle proposed by Chou and Talalay. The median effect dose of the drug combinations equation, Dm, r value, and combination index (CI) were calculated by CompuSyn software. A CI value below 1 represented synergism.

### 4.5. Colony Formation Assay

HepG2 cells (5 × 10^3^) and BEL7402 (3 × 10^4^) cells were plated on a 6-well plate. After incubation for 24 h, cells were treated with cisplatin, GRN A, and the combination of the two drugs at 37 °C under a humidified 5% CO_2_. New cultured media was replaced every 2 days; the cells were incubated for 14 days. Cells were stained with 0.05% crystal violet solution and visualized using a microscope. The number of colonies was determined by manual counting. Each of the experiments was performed more than three times. Data are expressed as means ± SD.

### 4.6. Construction of ENO1 Overexpression Cells 

ENO1 expression plasmids were synthesized in our laboratory, as described previously [25], and the plasmids were transferred into HepG2 and BEL7402 cells using lipofectamine 3000 (Thermo Fisher Scientific Inc., MA, USA),. as per the manufacture instruction. Briefly, HepG2 cells (5 ×10^3^) and BEL7402 (3 × 10^4^) cells were plated on a 96-well plate. After incubation for 24 h, ENO1 expression plasmids and lipofectamine 3000 (Thermo Fisher Scientific Inc., MA, USA) were added and incubated for 24 h. Cells with a high ENO1 expression were used to check their sensitivity to the co-treatment of cisplatin and GRN A.

### 4.7. Flow Cytometry Analysis 

Flow cytometry analysis was performed using the Annexin V FITC apoptosis detection kit (KeyGen Biotech, Ltd., Nanjing, China) as described previously [42]. Briefly, cells (1 × 10^5^) were incubated with cisplatin (33.33 μM), GRN A (14.53 μM) and the combination of the two drugs. After treatment for 24 h, cells were harvested by a centrifugation at 1000 g for 5 min, washed with phosphate-buffered saline (PBS) twice, and resuspended in 500 μL binding buffer (KeyGen Biotech, Ltd., Nanjing, China). Then, Annexin V FITC (5 μL) and PI (5 μL) were added. Cells were gently vortexed, incubated for 10 min at room temperature in the dark, and immediately analyzed by the FACS flow cytometry system (Bection-Dickinson, San Jose, CA, USA). Data are expressed as means ± SD; per group for at least 3 independent experiments.

### 4.8. Mitochondrial Membrane Potential Detection

HepG2 and BEL7402 cells were seeded to polystyrene tissue culture plates until ~80% confluency. Then the cells were treated with cisplatin, GRN A, and the combination of cisplatin and GRN A. After being treated for 24 h, the cells were washed gently with a PBS buffer and stained with a MITO-ID fluorescent probe and DAPI for 30 min in the dark. The cells were observed under a fluorescence microscope (Leica Microsystems Inc, IL, USA). The fluorescence intensities were measured using an Image Xpress Screening System (CA, USA).

### 4.9. Western Blot Analysis

Western blot analysis was performed as described previously [43]. Briefly, cells (1 × 10^6^) were seeded in a 60 mm culture dish. After incubation for 24 h, certain concentrations of cisplatin, GRN A, and the combined of cisplatin and GRN A were added and incubated for 48 h. Cells were harvested by centrifugation at 12,000 × *g* for 15 min at 4 °C, and the whole cell proteins were extracted using a whole cell protein extraction kit, according to the manufacturer’s instructions (Beyotime, lnc., Shanghai, China). The protein concentrations were determined using a BCA Protein Assay Kit (Thermo Fisher Scientific Inc., USA). Equal amounts of cellular lysates were subjected to sodium dodecyl sulfate (SDS) polyacrylamide gel electrophoresis and then transferred to 0.22 μm polyvinylidene difluoride (PVDF) membranes. After being blocked with a 5% bovine serum albumin (BSA) in TBST buffer (Tris-buffered saline, 0.1% Tween-20, and 5% nonfat dry milk powder), the membranes were incubated with the first antibodies overnight at 4 °C. After washing with PBS, the membranes were incubated with horseradish Peroxidase labeled secondary antibodies for 1 h at room temperature. Finally, protein bands were visualized by adding a Super Signal West Dura Extended Duration Substrate (Thermo Fisher Scientific Inc., MD, USA). The intensity of bands was quantified using ImageJ software (National Institutes of Health, Bethesda, MD, USA). All data were representatives of at least three independent experiments.

### 4.10. Tumor Xenograft Experiments

The animal experimental protocol was approved on 07 May 2018 by the Committee of Animal Experiments and Experimental Animal Welfare of Capital Medical University, protocol number AEEI-2018-081. Hepatocellular carcinoma cancer HepG2 cells (1 × 10^7^) were subcutaneously injected to the flanks of 6–8 weeks old female nude BALB/c mice. The mice were randomly divided into 4 groups; each group contained 5 mice. The control group was administrated through IP with saline containing 10% dimethyl sulfoxide (DMSO), while the cisplatin group was treated with cisplatin (5 mg/kg), and the GRN A group was treated with GRN A (50 mg/kg). The combination treated group was administrated with both cisplatin (5 mg/kg) and GRN A (50 mg/kg). All of the mice were recorded for 14 days. The tumor diameters were periodically measured with a caliper every two days. The volumes were calculated according to the following formula: Tumor Volume (mm^3^) = length × (width)^2^/2. No mice died of tumor loading in the experiments.

### 4.11. Immunohistochemistry

Tumors were removed after being recorded for 14 days. The major organs, including the heart, liver, spleen, lung, stomach, pancreas, and kidney were collected and fixed for the H&E staining analysis to elucidate a possible systematic toxicity. The excised tumor tissue and major organs section were fixed with 4% paraformaldehyde. These fixed samples were cut into 4–6 mm thick sections, embedded in paraffin, and were cut into 3-μm-thickserial sections. Major organs tissue sections were stained with H&E. Paraffin tumor sections were stained with anti-Ki-67 antibody using IHC staining to detect proliferation in tumor tissue. Images were taken with a high-capacity digital slide scanner system (3DHISTECH Ltd., Budapest, Hungary).

### 4.12. Statistical Analysis 

The data from the MTS assay were expressed as means ± SD. The IC_50_ values of cisplatin and GRN A were computed by GraphPad Prism 5 program (GraphPad Software Inc., San Diego, CA, USA). Dose-effect curve parameters, CI values were calculated by the CompuSyn program (Compusyn, Inc., Paramus, NJ, USA) and GraphPad Prism 5. Differences between the experimental groups and the control groups were analyzed using a student t-test. (t-test, *p < 0.05, **p < 0.01, ***p < 0.001)

## 5. Conclusions

In the present study, we found that GRN A, a peptide from the proteolysis of progranulin, possesses potent antitumor activity both in vitro and in vivo. GRN A is able to sensitize the antitumor effect of cisplatin in HCC cells, and the combination of GRN A and cisplatin is a promising strategy for the treatment of HCC tumors. Additionally, overexpression of ENO1 attenuates the synergistic effect of GRN A and cisplatin, suggesting that targeting ENO1 is a useful approach for enhancing the anticancer activity of cisplatin.

## Figures and Tables

**Table 1:** Granulin A (GRN A) enhanced the cytotoxicity of cisplatin in HepG2 cells. Cells were treated with cisplatin, cyclonphosphamide, paclitaxel, 5-fluorouracil, methotrexate, and gemcitabine, respectively, in the presence or absence of GRN A. After being treated for 48 h, the cell viability was determined by MTS assay, and IC_50_ values were calculated using SPSS10.0 software.

Chemotherapy Drugs	IC_50_ (μM)	IC_50_ (μM) Chemotherapy Drugs1plus GRN A (10 μM)
Cisplatin	99.54 ± 7.27	51.32 ± 5.41
Cyclophosphamide	33.50 ± 3.29	33.44 ± 3.86
Paclitaxel	1.54 ± 0.37	1.49 ± 0.17
5-Fluorouracil	96.51 ± 8.77	90.18 ± 6.52
Methotrexate	5.17 ± 0.47	4.64 ± 0.18
Gemcitabine	7.05 ± 0.93	6.25 ± 0.79

**Table 2:** The dose-effect relationship parameters and combination index (CI) values of cisplatin, GRN A, and the combination of cisplatin and GRN A.

	HepG2			BEL7402		
	Cisplatin	GRN A	Cisplatin Plus GRN A	Cisplatin	GRN A	Cisplatin Plus GRN A
Dm	53.45	68.42	3.90	82.62	179.92	18.44
r value	0.9847	0.9968	0.9883	0.9833	0.9776	0.9798
CI values at IC_50_	-	-	0.0618	-	-	0.1557

## Supplementary Materials

Supplementary materials can be found at http://www.mdpi.com/1422-0067/19/10/3060/s1.

## Abbreviations

MTS	3-(4,5-dimethylthiazol-2-yl)-5-(3-carboxymethoxyphenyl)-2-(4-sulfophenyl)-2H-tetrazolium
CI	Combination Index
IC_50_ Fa ENO1 GRN A IHC	Half maximal inhibitory concentration Fraction affected Alpha-enolase Granulin A Immunohistochemistry

## References

[1] Ghouri Y.A., Mian I., Rowe J.H. (2017). Review of hepatocellular carcinoma: Epidemiology, etiology, and carcinogenesis. J. Carcinog..

[2] Chen W., Zheng R., Baade P.D., Zhang S., Zeng H., Bray F., Jemal A., Yu X.Q., He J. (2015). Cancer statistics in China, 2015. CA Cancer J. Clin..

[3] Siegel R.L., Miller K.D., Jemal A. (2018). Cancer Statistics, 2018. CA Cancer J. Clin..

[4] Burkhart R.A., Ronnekleiv-Kelly S.M., Pawlik T.M. (2017). Personalized therapy in hepatocellular carcinoma: Molecular markers of prognosis and therapeutic response. Surg. Oncol..

[5] Bruix J., Gores G.J., Mazzaferro V. (2014). Hepatocellular carcinoma: Clinical frontiers and perspectives. Gut.

[6] Attwa M.H., El-Etreby S.A. (2015). Guide for diagnosis and treatment of hepatocellular carcinoma. World J. Hepatol..

[7] Dasari S., Tchounwou P.B. (2014). Cisplatin in cancer therapy: Molecular mechanisms of action. Eur. J. Pharmacol..

[8] Melnikov S.V., Soll D., Steitz T.A., Polikanov Y.S. (2016). Insights into RNA binding by the anticancer drug cisplatin from the crystal structure of cisplatin-modified ribosome. Nucleic Acids Res..

[9] Rutten H., Pop L.A.M., Janssens G.O.R.J., Takes R.P., Knuijt S., Rooijakkers A.F., van den Berg M., Merkx M.A., van Herpen C.M.L., Kaanders J.H.A.M. (2011). Long-Term Outcome and Morbidity after Treatment with Accelerated Radiotherapy and Weekly Cisplatin for Locally Advanced Head-and-Neck Cancer: Results of a Multidisciplinary Late Morbidity Clinic. Int. J. Radiat. Oncol. Biol. Phys..

[10] Zou W., Ma X., Hua W., Chen B., Cai G. (2015). Caveolin-1 mediates chemoresistance in cisplatin-resistant ovarian cancer cells by targeting apoptosis through the Notch-1/Akt/NF-κB pathway. Oncol. Rep..

[11] Zivanovic O., Abramian A., Kullmann M., Fuhrmann C., Coch C., Hoeller T., Ruehs H., Keyver-Paik M.D., Rudlowski C., Weber S. (2015). HIPEC ROC I: A phase I study of cisplatin administered as hyperthermic intraoperative intraperitoneal chemoperfusion followed by postoperative intravenous platinum-based chemotherapy in patients with platinum-sensitive recurrent epithelial ovarian cancer. Int. J. Cancer.

[12] Guan Y., Liu S., Wang H.-Y., Guo Y., Xiao W.-W., Chen C.-Y., Zhao C., Lu T.-X., Han F. (2016). Long-term outcomes of a phase II randomized controlled trial comparing intensity-modulated radiotherapy with or without weekly cisplatin for the treatment of locally recurrent nasopharyngeal carcinoma. Chi. J. Cancer.

[13] Hassan R., Sharon E., Thomas A., Zhang J., Ling A., Miettinen M., Kreitman R.J., Steinberg S.M., Hollevoet K., Pastan I. (2014). Phase 1 Study of the Antimesothelin Immunotoxin SS1P in Combination with Pemetrexed and Cisplatin for Front-Line Therapy of Pleural Mesothelioma and Correlation of Tumor Response with Serum Mesothelin, Megakaryocyte Potentiating Factor, and Cancer Antigen 125. Cancer.

[14] Basu P., Jenson A.B., Majhi T., Choudhury P., Mandal R., Banerjee D., Biswas J., Pan J., Rai S.N., Ghim S.J. (2016). Phase 2 randomized controlled trial of radiation therapy plus concurrent interferon-alpha and retinoic acid versus cisplatin for stage III cervical carcinoma. Int. J. Radiat. Oncol. Biol. Phys..

[15] Yeo W., Mok T.S., Zee B., Leung T.W.T., Lai P.B.S., Lau W.Y., Koh J., Mo F.K.F., Yu S.C.H.T. (2005). A randomized phase III study of doxorubicin versus cisplatin/interferon alpha-2b/doxorubicin/fluorouracil (PIAF) combination chemotherapy for unresectable hepatocellular carcinoma. J. Natl. Cancer Inst..

[16] Langer C.J., Gadgeel S.M., Borghaei H., Papadimitrakopoulou V.A., Patnaik A., Powell S.F., Gentzler R.D., Martins R.G., Stevenson J.P., Jalal S.I. (2016). Carboplatin and pemetrexed with or without pembrolizumab for advanced, non-squamous non-small-cell lung cancer: A. randomised, phase 2 cohort of the open-label KEYNOTE-021 study. Lancet Oncol..

[17] Manohar S., Leung N. (2018). Cisplatin nephrotoxicity: A review of the literature. J. Nephrol..

[18] Alhadeff A.L., Holland R.A., Nelson A., Grill H.J., De Jonghe B.C. (2015). Glutamate Receptors in the Central Nucleus of the Amygdala Mediate Cisplatin-Induced Malaise and Energy Balance Dysregulation through Direct Hindbrain Projections. J. Neurosci..

[19] Eiseman J.L., Beumer J.H., Rigatti L.H., Strychor S., Meyers K., Dienel S., Horn C.C. (2015). Plasma pharmacokinetics and tissue and brain distribution of cisplatin in musk shrews. Cancer Chemother. Pharmacol..

[20] Kottschade L., Novotny P., Lyss A., Mazurczak M., Loprinzi C., Barton D. (2016). Chemotherapy-induced nausea and vomiting: Incidence and characteristics of persistent symptoms and future directions NCCTG N08C3 (Alliance). Support. Care Cancer.

[21] Larkin J., Ascierto P.A., Dreno B., Atkinson V., Liszkay G., Maio M., Mandala M., Demidov L., Stroyakovskiy D., Thomas L. (2014). Combined Vemurafenib and Cobimetinib in BRAF-Mutated Melanoma. N. Engl. J. Med..

[22] Thatcher N., Hirsch F.R., Luft A.V., Szczesna A., Ciuleanu T.E., Dediu M., Ramlau R., Galiulin R.K., Balint B., Losonczy G. (2015). Necitumumab plus gemcitabine and cisplatin versus gemcitabine and cisplatin alone as first-line therapy in patients with stage IV squamous non-small-cell lung cancer (SQUIRE): An open-label, randomised, controlled phase 3 trial. Lancet Oncol..

[23] Tolkatchev D., Malik S., Vinogradova A., Wang P., Chen Z., Xu P., Bennett H.P.J., Bateman A., Ni F. (2008). Structure dissection of human progranulin identifies well-folded granulin/epithelin modules with unique functional activities. Protein Sci..

[24] Wang X., Xu H., Chen X., Tian Y., Wang F., Lin X. (2016). Cloning, expression and cytotoxicity of granulin A., a novel polypeptide contained in human progranulin. Biosci. Trends.

[25] Chen X., Xu H., Wu N., Liu X., Qiao G., Su S., Tian Y., Yuan R., Li C., Liu X. (2017). Interaction between granulin A and enolase 1 attenuates the migration and invasion of human hepatoma cells. Oncotarget.

[26] Nishimura K., Tsuchiya Y., Okamoto H., Ijichi K., Gosho M., Fukayama M., Yoshikawa K., Ueda H., Bradford C.R., Carey T.E. (2014). Identification of chemoresistant factors by protein expression analysis with iTRAQ for head and neck carcinoma. Br. J. Cancer.

[27] Qian X., Xu W., Xu J., Shi Q., Li J., Weng Y., Jiang Z., Feng L., Wang X., Zhou J. (2017). Enolase 1 stimulates glycolysis to promote chemoresistance in gastric cancer. Oncotarget.

[28] Yeo M.-K., Kim H.E., Kim S.H., Chae B.J., Song B.J., Lee A. (2017). Clinical usefulness of the free web-based image analysis application ImmunoRatio for assessment of Ki-67 labelling index in breast cancer. J. Clin. Pathol..

[29] Lee Y.J., Lee S., Ho J.N., Byun S.-S., Hong S.K., Lee S.E., Lee E. (2014). Synergistic antitumor effect of ginsenoside Rg3 and cisplatin in cisplatin-resistant bladder tumor cell line. Oncol. Rep..

[30] Zhang Y., Zheng S., Zheng J.-S., Wong K.-H., Huang Z., Ngai S.-M., Zheng W., Wong Y.-S., Chen T. (2014). Synergistic Induction of Apoptosis by Methylseleninic Acid and Cisplatin, The Role of ROS-ERK/AKT-p53 Pathway. Mol. Pharm..

[31] Xia A., Li H., Li R., Lu L., Wu X. (2018). Co-treatment with BEZ235 enhances chemosensitivity of A549/DDP cells to cisplatin via inhibition of PI3K/Akt/mTOR signaling and downregulation of ERCC1 expression. Oncol. Rep..

[32] Hsu C.-M., Lin P.-M., Tsai Y.-T., Tsai M.-S., Tseng C.-H., Lin S.-F., Yang M.-Y. (2018). NVP-BEZ235, a dual PI3K-mTOR inhibitor, suppresses the growth of FaDu hypopharyngeal squamous cell carcinoma and has a synergistic effect with Cisplatin. Cell Death Dis..

[33] Bhandari V., Bateman A. (1992). Structure and Chromosomal Location of the Human Granulin Gene. Biochem. Biophys. Res. Commun..

[34] Bateman A., Bennett H.P.J. (1998). Granulins: The structure and function of an emerging family of growth factors. J. Endocrinol..

[35] Culouscou J., Carlton G., Shoyab M. (1993). Biochemical-Analysis of the Epithelin Receptor. J. Biol. Chem..

[36] Shoyab M., McDonald V.L., Byles C., Todaro G.J., Plowman G.D. (1990). Epithelins 1 and 2: Isolation and characterization of two cysteine-rich growth-modulating proteins. Proc. Natl. Acad. Sci. USA.

[37] Fu Q.-F., Liu Y., Fan Y., Hua S.-N., Qu H.-Y., Dong S.-W., Li R.-L., Zhao M.-Y., Zhen Y., Yu X.-L. (2015). Alpha-enolase promotes cell glycolysis, growth, migration, and invasion in non-small cell lung cancer through FAK-mediated PI3K/AKT pathway. J. Hematol. Oncol..

[38] Leonard P.G., Satani N., Maxwell D., Lin Y.-H., Hammoudi N., Peng Z., Pisaneschi F., Link T.M., Lee G.R., Sun D. (2016). SF2312 is a natural phosphonate inhibitor of enolase. Nat. Chem. Biol..

[39] Jung D.-W., Kim W.-H., Park S.-H., Lee J., Kim J., Su D., Ha H.-H., Chang Y.-T., Williams D.R. (2013). A Unique Small Molecule Inhibitor of Enolase Clarifies Its Role in Fundamental Biological Processes. A. Chem. Biol..

[40] Aston W.J., Hope D.E., Nowak A.K., Robinson B.W., Lake R.A., Lesterhuis W.J. (2017). A systematic investigation of the maximum tolerated dose of cytotoxic chemotherapy with and without supportive care in mice. BMC Cancer.

[41] Chou T., Tan Q., Sirotnak F. (1993). Quantitation of the synergistic interaction of edatrexate and cisplatin in vitro. Cancer Chemother. Pharmacol..

[42] Wei C., Chen C., Cheng Y., Zhu L., Wang Y., Luo C., He Y., Yang Z., Ji Z. (2018). Ailanthone induces autophagic and apoptotic cell death in human promyelocytic leukemia HL-60 cells. Oncol. Lett..

[43] Yuan R., Xu H., Liu X., Tian Y., Li C., Chen X., Su S., Perelshtein I., Gedanken A., Lin X. (2016). Zinc-Doped Copper Oxide Nanocomposites Inhibit the Growth of Human Cancer Cells through Reactive Oxygen Species-Mediated NF-κB. Activations. ACS Appl. Mater. Interfaces.

