# Supplementary material for: Granulin A Synergizes with Cisplatin to Inhibit the Growth of Human Hepatocellular Carcinoma
Source: Int J Mol Sci. 2018 Oct 7;19(10):3060. doi: 10.3390/ijms19103060 (PMC6213591; doi:10.3390/ijms19103060)
Supplement: Supplementary file 1 [file ijms-19-03060-s001.docx]

**Supplementary Materials**

**
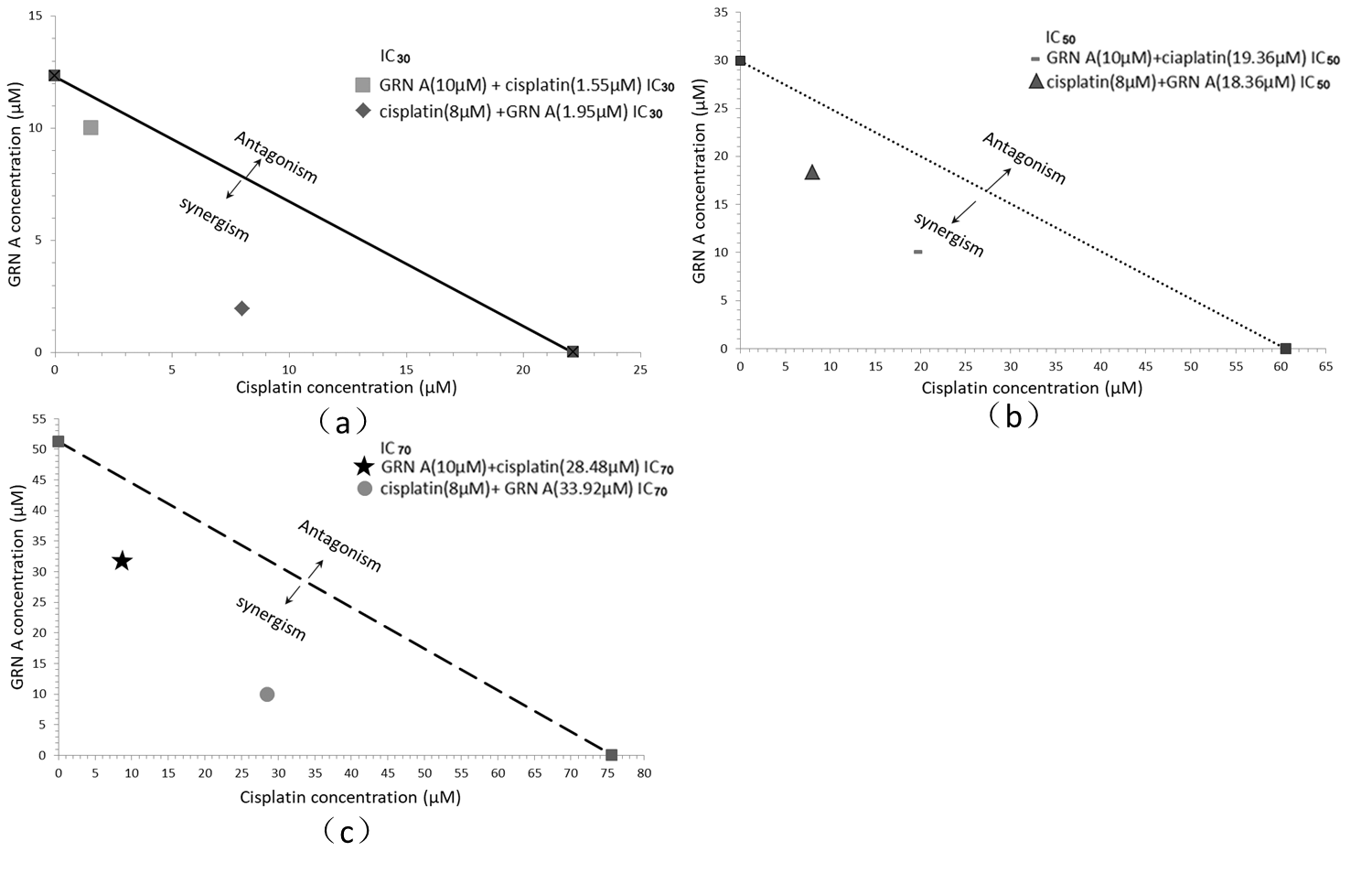
**

**Figure S1.** Isobolograms of combined cisplatin and GRN A in HepG2 cells. (a) The drug concentration needed to reach IC_30_. When treating the cells with 10μM GRN A, 1.55μM cisplatin was needed (
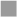
). Treating the cells with 8μM cisplatin, 1.95μM GRN A was needed (
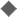
). (b) The drug concentration needed to reach IC_50_. Treating the cells with 10μM GRN A, 19.36μM cisplatin was needed (

). Treating the cells with 8μM cisplatin, 18.36μM GRN A was needed (
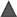
). (c) The drug concentration needed to reach IC_70_. Treating the cells with 10μM GRN A, 28.48μM cisplatin was needed (
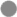
). Treating the cells with 8μM cisplatin, 33.92μM GRN A was needed (
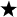
).
